# Crystal Structure of the Carboxy-Terminal Region of the Bacteriophage T4 Proximal Long Tail Fiber Protein Gp34

**DOI:** 10.3390/v9070168

**Published:** 2017-06-30

**Authors:** Meritxell Granell, Mikiyoshi Namura, Sara Alvira, Shuji Kanamaru, Mark J. van Raaij

**Affiliations:** 1 Departmento de Estructura de Macromoleculas, Centro Nacional de Biotecnologia (CNB-CSIC), Calle Darwin 3, E-28049 Madrid, Spain; 2 Department of Life Science and Technology, Tokyo Institute of Technology, M6-11 2-12-1 Ookayama, Meguro-ku Tokyo 152-8550, Japan; 3ki44xyz@gmail.com; 3 Departmento Bioquimica y Biologia Molecular, Facultad de Farmacia, Universidad de Santiago de Compostela, E-15782 Santiago de Compostela, Spain

**Keywords:** bacterial viruses, *Caudovirales*, *Myoviridae*, crystallography, fibrous protein

## Abstract

Long tail fibers of bacteriophage T4 are formed by proteins gp34, gp35, gp36, and gp37, with gp34 located at the phage-proximal end and gp37 at the phage-distal, receptor-binding end. We have solved the structure of the carboxy-terminal region of gp34, consisting of amino acids 894–1289, by single-wavelength anomalous diffraction and extended the structure to amino acids 744–1289 using data collected from crystals containing longer gp34-fragments. The structure reveals three repeats of a mixed α-β fibrous domain in residues 744 to 877. A triple-helical neck connects to an extended triple β-helix domain (amino acids 900–1127) punctuated by two β-prism domains. Next, a β-prism domain decorated with short helices and extended β-helices is present (residues 1146–1238), while the *C*-terminal end is capped with another short β-helical region and three β-hairpins. The structure provides insight into the stability of the fibrous gp34 protein.

## 1. Introduction

Bacteriophages (or phages) are of interest as tools to understand principles of molecular biology [1], vectors of horizontal gene transfer [2], drivers of bacterial evolution [3], sources of diagnostic and genetic tools, but also as novel therapeutic agents [4]. Bacteriophages recognize bacteria specifically and infect them efficiently. The great majority of known phages belong to the *Caudovirales* order of double-stranded DNA phages [5,6]. These phages have a tail that recognizes and attaches to the host and subsequently orchestrates DNA transfer into the bacterial cytoplasm. Bacteriophage T4 infects *Escherichia coli*. It is a complex tailed virus and belongs to the *Myoviridae* family, with an exclusively lytic lifecycle [7]. The mature virus consists of a prolate head encapsulating the genomic DNA and a cylindrical contractile tail. 

The tail of bacteriophage T4 is a large macromolecular complex [8,9]. It consists of a sheath, an internal tail tube, and a baseplate, which is situated at the distal end. The tail has two types of fibers, the long tail fibers and the short tail fibers, responsible for host cell recognition and binding to the host cell. These fibers are attached to the baseplate. In free T4 phages, most of the long tail fibers are retracted, folded back against the phage, although some are extended and free to contact bacterial cells [9,10]. The long tail fibers recognize the outer membrane protein C (OmpC) or the lipopolysaccharide (LPS) of *E. coli* and are responsible for the initial and reversible attachment of the virion. After several long tail fibers have bound, the baseplate changes conformation and the short tail fibers, which normally are incorporated into the baseplate, extend and bind irreversibly to the host cell LPS [9,11]. The short tail fibers serve as inextensible stays during contraction of the tail sheath and penetration of the cell envelope by the tail tube [10]. 

**Figure 1:**
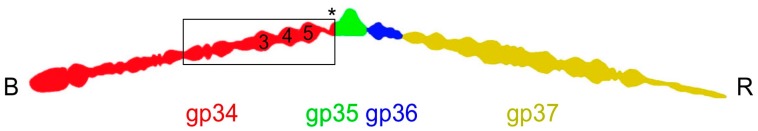
Schematic drawing of the bacteriophage T4 long tail fiber. The baseplate-binding proximal end is indicated with a B, the receptor-binding distal end with an R. The component proteins are indicated with colors: gp34 in red, gp35 in green, gp35 in blue, and gp37 in yellow. The carboxy-terminal end of gp34 is indicated with an asterisk. The numbers inside the red gp34 outline indicate where the P3, P4, and P5 domains are located. A box indicates the approximate size of the largest fragment for which the structure was solved (residues 744–1289).

The long tail fibers of bacteriophage T4 are connected to the baseplate via gp9 [9,12]. When retracted, they also contact the fibritin (whisker) proteins of the neck region between the head and the tail of the phage and the capsid. The fibers are about 145 nm long and only 4 nm in diameter. Each fiber consists of rigid proximal and distal parts, connected by a hinge region (Figure 1) [13,14]. The proximal half-fiber is formed by a parallel homo-trimer of gp34. The carboxy-terminal end of gp34 interacts with the distal half-fiber; the hinge angle between the proximal and distal half-fibers may be due to the monomeric protein gp35. Gp35 forms the “knee-cap”, by which the retracted long tail fibers bind to the whiskers. The gp36 trimer is located near gp35, while gp37 makes up the rest of the shin, including the very distal receptor-recognizing tip [8,10,15].

The large fibrous proteins gp34 (1289 residues) and gp37 (1026 residues) need specialized chaperone proteins to help them fold and trimerize correctly. Gp57 appears to be a general T4 tail fiber chaperone and is needed for the correct trimeric assembly of gp12 (the protein forming the short tail fibers), gp34, and gp37 [16,17]. Gp38 must also be present for the correct trimeric assembly of gp37 [16,18]. The molecular basis of the gp38 and gp57 chaperone activities are unclear, but gp57 may function to keep fiber protein monomers from aggregating unspecifically, while gp38 might bring together three carboxy-terminal ends of the gp37 monomers to start the folding process. The long tail fibers appear to be stiff structures, because no kinked half-fibers have been observed in electron micrographs; moreover, the angle between the half fibers in the complete fiber never deviates much from 20° [13]. The stiffness may be necessary for transmitting the receptor recognition signal from the tip of the fiber to the baseplate [8]. Of the T4 tail fibers, only the crystallographic structures of the carboxy-terminal half of the short tail fibers [19,20] and the tip of gp37 are known [15], and the structure of the entire short tail fiber has been modelled into the pre-attachment state of the baseplate at around 4 Å resolution [9].

In the current work, by co-expression with gp57, we have produced and crystallized carboxy-terminal fragments of the proximal tail fiber protein gp34 [14]. We have solved the structure of a trimeric fragment containing amino acids 894 to 1289 (the carboxy-terminus) by single-wavelength anomalous diffraction and, by molecular replacement, a larger fragment containing residues 744 to 1289. The overall structure reveals three small domains containing three parallel α-helices each, followed by triple β-helix regions punctuated by β-stranded globular domains.

## 2. Materials and Methods 

Gp34(726–1289), gp34(781–1289), and gp34(894–1289) were produced, crystals were obtained, and data was collected from them as described [14]. For the gp34(894–1289) crystals containing selenomethionine, the coordinates of the heavy atom sites were obtained using Shelxd [21] and initial phases were determined using Autosharp [22]. Solvent flattening was performed with Solomon [23]. ARP/wARP was used for automatic model building to 2.0 Å resolution [24]. For the remaining structures, molecular replacement was done with Phaser [25] or Molrep [26]. Model building was performed with Coot [27] and refinement with Refmac [28] or with Phenix [29]. Structure validation was carried out using Molprobity [30] and the Protein Data Bank (PDB) validation service [31]. Structure figures were prepared with Pymol [32] or Chimera [33]. Structure comparisons were done with Dali [34], oligomerization parameters (accessible and buried surfaces, estimated dissociation energies) were analyzed with Jspisa [35] and local three-fold axes were identified with Symd [36]. Multiple amino acid sequences were aligned by Clustal W [37].

## 3. Results

### 3.1. Structure Solution

Crystals belonging to two different space groups (*P*2_1_ and *R*32) were obtained for gp34(894–1289). Crystals of gp34(781–1289) and gp34(726–1289) also belong to space group *P*2_1_, but with different cell parameters. Statistics of data collected from these crystals are shown in Table 1. The structure of gp34(894–1289) was determined using the single-wavelength anomalous dispersion method (SAD), using data from crystals of the *P*2_1_ form grown from protein in which selenomethionine was incorporated. A high-multiplicity dataset measured to 2.0 Å resolution at the peak wavelength of selenium was collected and used for phase determination. Thirteen sites were found, where fifteen sites were expected (five internal methionines per monomer of the trimer). The resulting phases were of high quality (Table 1) and, after solvent flattening with 53.2% solvent, permitted automatic building of 1102 residues, of which 1095 were automatically docked into sequence. Manual fitting and refinement led to a final model consisting of 1189 residues (893–1288 of chain A, 893–1289 of chain B, and 893–1288 of chain C), plus seven glycerol molecules and 1620 water molecules (residue 893 is the final proline of the amino-terminal purification tag). The obtained model was also refined against data collected to 2.0 Å resolution from a native crystal (i.e., not modified with selenomethionine) of the same crystal form (*P*2_1_ space group), using the same test selection set for calculating the free R-factor selected in thin resolution shells. Here, the refined model contained the same protein residues as before, plus seven glycerol molecules and 1312 water molecules. Both the native and selenomethionine-modified models have good correspondence to the measured data and reasonable stereochemical parameters (Table 1), although residues at the amino- and carboxy-termini have higher temperature factors and show some disorder.

The structure of the *R*32 crystal form was solved by molecular replacement, using the hexagonal setting *H*32. Four trimers (i.e., twelve identical protein chains) were found in the asymmetric unit. Here, manual fitting and refinement led to a final model consisting of 4736 residues (894–1288 of chain A, 894–1288 of chain B, 890–1287 of chain C, 893–1287 of chain D, 890–1287 of chain E, 893–1288 of chain F, 894–1288 of chain G, 894–1287 of chain H, 895–1288 of chain I, 895–1288 of chain J, 897–1287 of chain K, and 894–1288 of chain L). For final refinement at 3.0 Å resolution, secondary structure restraints were used as without them the model tended to get distorted. No solvent molecules were included.

The structures of gp34(781–1289) and gp34(726–1289) were also solved by molecular replacement, both with one trimer in the asymmetric unit and belonging to the *P*2_1_ space group. After manual adjustments and refinement, the gp34(781–1289) structure contained 1485 residues (795–1289 in each of the three chains), plus 3 phosphate ions, 17 glycerol molecules, 1822 water molecules, three phosphate ions, a urea molecule, and two acetate ions. Note that this structure contains selenomethionine residues instead of methionine, although these were not used for phasing. The gp34(726–1289) structure was composed of 1668 residues (744–1289 in each of the three chains), a glycerol, and 350 water molecules. The *N*-termini of gp34(726–1289) and gp34(781–1289) are less well ordered in the crystal structures, presumably due to flexibility and lack of crystal contacts in these regions.

### 3.2. Overall Structure

**Figure 2:**
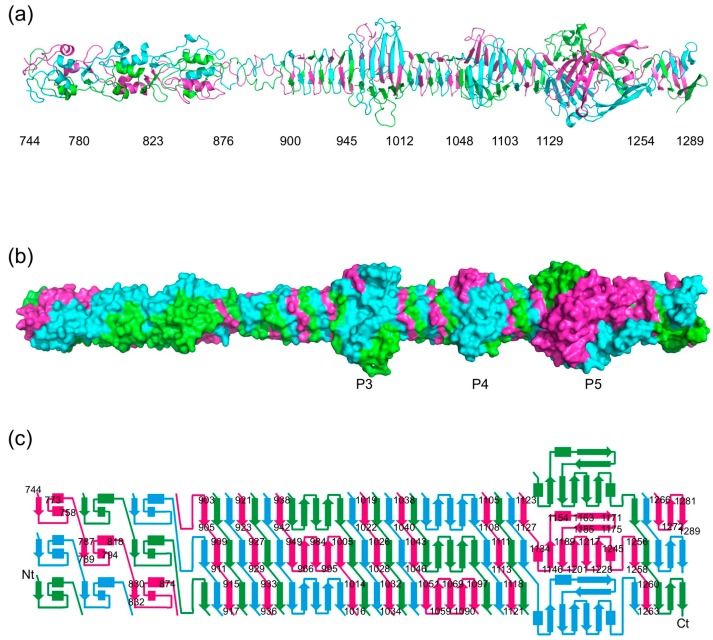
Crystallographic structure of the carboxy-terminal part of gp34. (**a**) Cartoon view of the structure of gp34(744–1289). Approximate domain boundaries are indicated. (**b**) Space-filled representation of the structure in the same orientation as A, illustrating the extensive intertwining of the three monomers in the trimer. (**c**) Topology diagram of the structure. The termini, as well as the starts and ends of most of the secondary structure elements, are labelled.

The structure of the carboxy-terminal part of gp34 reveals an elongated protein; just over 30 nm long for the longest crystallographic model, containing residues 744–1289 (Figure 2). The carboxy-terminal parts of gp34 that we crystallized occupy the part of the proximal tail fiber closest to gp35 (the “knee”). The overall shape of the molecule fits very well with the corresponding region of the averaged electron microscopy image produced by Cerritelli et al. [13].

Residues 796–878 form two domains, each with three central short α-helices decorated by intertwined loops. They are followed by a triple-helical neck and a stretch of residues in which two turns are present. The framework of the rest of the structure (residues 901 to 1270) is an 18 nm-long triple β-helix made up of parallel β-strands. The widths in the triple β-helix regions are around 2 nm. The triple β-helix is interspersed with three wider domains, the widest of which reaches just over 5 nm in diameter. These domains are named P3, P4, and P5 according to the nomenclature coined by Cerritelli et al. [13] (Figure 1). The cores of each of these three domains are formed by three anti-parallel β-sheets, one contributed by each of the three monomers. In the case of P3 and P4, the first and last β-strands of their anti-parallel β-sheets stack exactly onto the triple β-helices preceding and following them, while for P5 this is not the case. At the carboxy-terminal end of the P5 structure, the triple β-helix stacks onto a final three-stranded anti-parallel β-sheet. These last strands appear somewhat more flexible, as evidenced by poorer electron density and higher temperature factors, and may be stabilized, or even change their conformation, in the presence of gp35 and/or gp36. Between the start and the end of the structure, each of the three protein chains makes seven 360-degree turns around the trimer axis.

### 3.3. Stability of the Trimer

In the formation of the trimer, more than half of the total accessible surface of the isolated monomer is buried (23.5 × 103 Å^2^ of 43.8 × 103 Å^2^ for residues 744–1289, 21.4 × 103 Å2 of 39.5 × 103 Å^2^ for amino acids 795–1289, and 17.2 × 103 Å^2^ of 31.6 × 103 Å^2^ for residues 894–1289). The estimated energies necessary to dissociate the complexes are 660, 608, and 488 kcal/mol for residues 744–1289, 795–1289, and 894–1289, respectively (estimated using Jspisa [35]). The extensive intertwining of the three monomers in the trimer leads to a large fraction of buried monomer surface in the trimer, a large amount of inter-molecular hydrophobic and polar interactions, and thus a very high energy barrier to dissociation. This predicted stability is consistent with the observed heat and protease stability of gp34(781–1289) [14].

### 3.4. The α-Helix-Containing Domains

**Figure 3:**
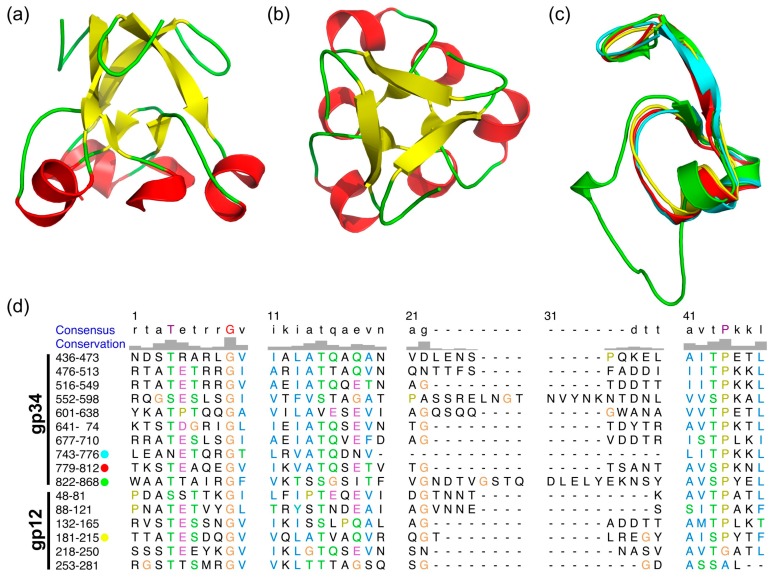
Structure comparison of *N*-terminal domains and repeating sequences of gp34. (**a**) Side view of the trimeric structure of one of the amino-terminal domains, residues 745–776. (**b**) Top view of (a). (**c**) Superposition of the monomeric structure of the three amino-terminal repeats of gp34(744–776 in cyan, 780–812 in red, 823–868 in green) and one repeat of gp12(182–215) in yellow. (**d**) Alignment of repeating sequences of gp34 and gp12.

As previously reported [13,19], gp34 contains repeating sequences (Figure 3c), of which three repeats are resolved in our structure. Structure based sequence alignment shows the repeating sequence to be longer than reported and reveals an additional repeat from residue 552 to 598. These repeats form a domain which starts from a tight turn at consensus position 1 to 8 towards the three-fold axis of the trimer, followed by a β-strand at position 9 to 14 (Figure 3). This strand points to the outside of the molecule and connects to an α helix at position 16 to 20. A non-conserved loop at positions 21 to 40, which is different in length in different repeats, connects back to the central core of the domain and forms a short β strand at position 41 and 42. Consensus residues 44 to 47 form an α-helix, leading to a three-helix bundle at the three-fold symmetry axis. In the repeat consisting of residues 822–868, the non-conserved loop (amino acids 837–859) points upwards, forming a 2-nm long β-hairpin lying against the three-helix bundle.

As shown in Figure 3d, these repeats are also present in the T4 short tail fiber protein gp12, of which the structure in the T4 native baseplate was reported [9]. Gp34 residues 744–776, 780–812, 823–868, and gp12 182–215 can be superimposed nicely (Figure 3c), and the repeating sequences are well-conserved. Especially well-conserved is the glycine at position 9, which makes a 90° turn and starts the β-strand. Positions 43 (Ser or Thr) and 44 (Pro) are also well-conserved at the *N*-terminal end of the central α-helix. Ser or Thr are favorable at the N-1 (Ncap) position of α-helices and Pro is commonly found at the N-terminus of α-helices [38].

Residues 879–889 form a tight triple helix, followed by a stretch of residues (890–900) in which two turns are present. This region connects the repeat domain to the triple β-helix domain discussed below.

### 3.5. The Triple β-Helix

Amino acids 901–944, 1014–1047, 1105–1128, and 1255–1271 of the carboxy-terminal part of gp34 form a triple β-helix (Figure 2a). It is composed of intertwined β-strands, which are nearly perpendicular to the fiber axis. It has exclusively inter-monomer main-chain hydrogen bonds and its hydrophobic core has a triangular cross-section. The triple β-helix is reminiscent of the long β-helix in gp5 (gp5 is the tail lysozyme of phage T4, which functions as a cell-puncturing device during infection [39]). However, the gp34 triple β-helix is less wide and has a hydrophobic interior, more like the short triple β-helix domain in the T4 short tail fiber (gp12) [19]. As for gp12, the width is about 2 nm, compared to over 3 nm for the gp5 triple β-helix. The repetitive structure of the triple β-helix is not expressed in clear sequence motifs, although some generalizations may be made. Aliphatic residues like Leu and Ile are abundant in the β-strands, forming the hydrophobic core, meanwhile small residues like Gly, Ser, and Asn are abundant in the loops, allowing for the turns between the strands. A rare, but very clearly resolved, non-proline *cis*-peptide [40] is present between Asn1121 and Ser1122 at the end of one of the β-strands of the triple β-helix.

At the carboxy-terminal end, a mixed five-stranded β-sheet from each monomer caps the gp34 structure. The first three strands are parallel, while the last three are anti-parallel. The three sheets from each monomer pack together, enclosing a hydrophobic core. In the crystal structures, the carboxy-terminal ends show some disorder and higher temperature factors. As this is the region that most likely interacts with gp35 or/and gp36 in the complete long tail fiber, it is possible it becomes more ordered and even changes conformation upon incorporation of gp35 and/or gp36.

### 3.6. The P3, P4, and P5 Domains

**Figure 4:**
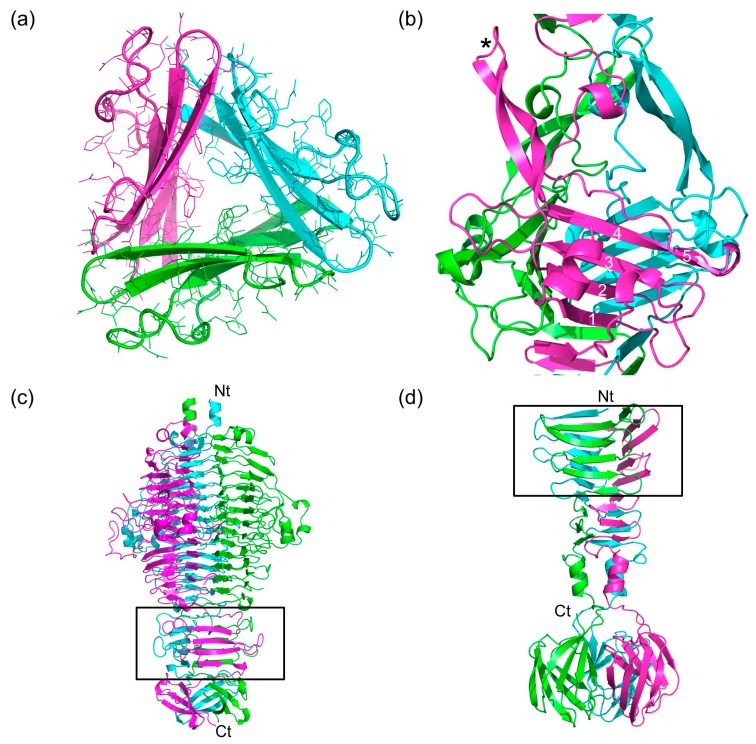
Triangular domains in bacteriophage fibers and tailspikes. (**a**) Cross-section of the P4 domain, viewed from the *N*-terminal end. (**b**) Side view of the P5 domain. One of the β-hairpins is indicated with an asterisk. The β-strands of the central β-sheet are numbered. Note that the fifth strand is irregular. (**c**) Side view of the bacteriophage P22 tailspike, which is a trimer of gp9. The triangular domain in this and the next panel are boxed and the termini are labelled. (**d**) Side view of the *C*-terminal part of the bacteriophage T7 tail fiber (gp17). In panels (**c**) and (**d**), *N*- and *C*-termini are labelled with Nt and Ct, respectively.

The P3, P4, and P5 domains are wider and protrude sideways from the triple β-helix. The widths of domains P3 and P4 are around 4.5 nm and 3.8 nm, respectively (Figure 4a). Each of the three domains has an anti-parallel five-stranded β-sheet at its core. Three equivalent β-sheets form a triangular and hydrophobic core. The P3 domain has short loops between the five strands of its β-sheet, except for the loops between the first and second strand and between the second and third strand. The P4 domain also has a five-stranded anti-parallel β-sheet at its core, in this case, only the loop between the third and fourth strands is longer. This loop folds back towards the amino-terminal direction of the protein, covering part of strands 1, 2, and 3. The β-sheets of the P3 and P4 domains stack onto the β-strands of the triple β-helices preceding and following them, forming a 27-strand longitudinal mixed β-sheet (parallel in the triple β-helix regions and anti-parallel in the P3 and P4 domains).

Tailspikes from phages like the podovirus P22 (Protein Data Bank (PDB) entry 1TSP; [41]) (Figure 4c) and the myovirus Det7 (PDB entry 2V5I, [42]) contain trimeric anti-parallel β-sheets in their triangular domains that are similar to those in the P3 and P4 domains. Triangular domains are also present in the side tail fibers of the podovirus T7 (PDB entry 4A0T, [43]) (Figure 4d) and the siphovirus T5 (PDB entry 4UW7, [44]). However, there are no exact structural homologues of the P5 domain in known trimeric phage fibers or tailspikes, so this domain has a novel fold. The P5 domain is the largest and is 5.1 nm wide. Its framework is an anti-parallel β-sheet like in the P3 and P4 domains, but the fifth strand is irregular and the loops connecting the strands are longer and more elaborate (Figure 4b). The core is also not completely hydrophobic and has ordered solvent molecules inside.

Between the first and second strands of the P5 domain, a long loop including a short α-helix and a β-hairpin is inserted, which extends about 3.5 nm towards the carboxy-terminal end of the structure. The β-hairpin motif is reminiscent of the β-hairpin arms in tailspike chaperones of K1F (PDB entry 3GW6; [45]), phi29 (PDB entry 3SUC; [46]) and pb1 (PDB entry 4UW8; [44]) and may serve a similar role here, promoting the correct folding and trimerization of the structure they enclose. Given the size of the P5 domain and the fact that it is the domain of gp34 with most inter-monomer contacts, folding and trimerization of gp34 may start with this domain.

### 3.7. Fitting of the Crystal Structure in an EM Map of T4 Phage

**Figure 5:**
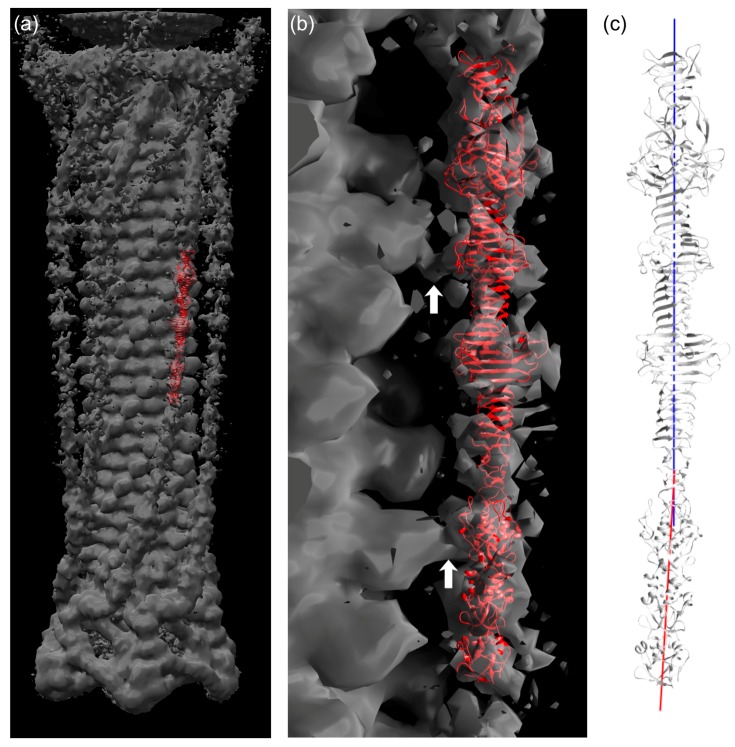
Fitting of the gp34(744–1289) crystal structure in cryo-electron microscopy density of the bacteriophage T4 tail. (**a**) Crystal structure in red fitted into EM density of the T4 tail (EMD-1126, grey). (**b**) Close view of (a). White arrows indicate connected EM density between long tail fiber and phage sheath. (**c**) Representation of local three-fold axis of the *C*-terminal part (blue, 882–1289) and *N*-terminal part (red, 744–881).

The longest structure, gp34(744–1289), was fitted into the reconstructed electron microscopy (EM) map of the extended T4 tail [47]. Although the density for the long tail fiber is not as clear as for the other parts of the tail, it was possible to fit the crystal structure manually into the density by using domains P3, P4, and P5 as an indicator (Figure 5a,b). As shown in Figure 5, the crystal structure is slightly bent around residue 881. The EM density for the gp34 fiber is also curved. Thus, we think the bending is a feature of this protein.

When the contour level of the EM density is increased, at least three connecting densities between the long tail fiber and the tail sheath become visible. The first site is located around the loop containing residues 1076–1087 (Figure 5b, top arrow). The second one is found at the long insertion in one of the repeating structures (residues 836–857; Figure 5b, lower arrow). Although both the loop and the insertion are folded against the rest of gp34 in the crystal structure, it is conceivable that, in the phage particle, they might protrude from trimer and interact with the tail sheath protein gp18. This might help the tail fiber to assemble to the phage tail. The third connecting density is located close to the *N*-terminus of gp34, for which a crystal structure is not available. However, the fourth repeating sequence from the *N*-terminus, residue 552–598, also has an insertion (Figure 3d). Thus, it is possible that the long insertion in the fourth repeating sequence is responsible for the third connected density.

## 4. Discussion

We have solved the structure of the carboxy-terminal third of the proximal long tail fiber protein gp34. The protein is a highly intertwined trimer containing a 2 nm-wide triple β-helix domain interspersed with three wider domains in which each of the monomers forms an anti-parallel β-sheet. The third of these domains features nearly 4 nm-long “arms” that fold against the carboxy-terminal part of the structure. From the *N*-terminus to the triple β-helix, there are several repeats of an α-helix containing motif, which are also present in the short tail fiber protein gp12. These structural repeats expand the folds known for fibrous proteins, which in turn may have benefits for the design of protein-based materials [48,49].

The extensive intermolecular interactions between the three protein chains shed light on the stability of the protein. The structure of gp34 also illustrates how phages use similar domains in different proteins, mixing, matching, and duplicating domains in their evolution until a tailspike or fiber is obtained that initiates the infection process efficiently. Future studies should reveal high-resolution structural information on the amino-terminal part of gp34 and on how the trimeric gp34 carboxy-terminus interacts with the gp35 “knee-cap” monomer and the distal part of the tail fiber.

## Figures and Tables

**Table 1:** Crystallographic data, phasing, and refinement statistics.

Data Collection					
	*P*2_1_-SeMet (894–1289)	*P*2_1_-native (894–1289)	*H*32-native (894–1289)	*P*2_1_-SeMet (781–1289)	*P*2_1_-native (726–1289)
Cell axes (a, b, c, Å)	92.8, 76.3, 117.0	93.0, 76.1, 116.8	228.5, 228.5, 1069.5	92.1, 75.9, 149.1	107.3, 76.1, 139.9
Cell angles (α, β, γ, °)	90.0, 99.3, 90.0	90.0, 99.1, 90.0	90.0, 90.0, 120.0	90.0, 90.2, 90.0	90.0, 97.6, 90.0
Beamline	Diamond I04	ESRF ID14-1	ESRF ID14-4	PF, BL1A	PF, BL17A
Resolution range (Å)	30–2.0 (2.11–2.00) *	30–2.0 (2.11–2.00)	30–3.0 (3.16–3.00)	46.1–1.9 (2.00–1.90)	45.2–2.89 (3.04–2.89)
Reflections	109,012 (15,872)	108,802 (15,849)	214,066 (31,052)	161,501 (23,029)	48930 (6784)
Multiplicity	7.4 (7.5)	3.4 (3.4)	4.6 (4.7)	4.9 (4.9)	3.3 (3.3)
Completeness (%)	99.9 (99.9)	99.9 (99.9)	99.8 (99.8)	99.0 (97.0)	96.6 (91.8)
Mean <I/s(I)>	12.4 (5.6)	8.1 (3.3)	8.4 (3.5)	10.0 (3.2)	9.4 (3.0)
R_merge_ (%) ^†^	11.1 (30.7)	10.6 (37.7)	12.9 (36.1)	11.5 (42.5)	9.8 (43.6)
Wilson B (Å^2^)	13.7	14.9	55.6	15.0	69.5
Phasing					
Heavy atom sites ^‡^	13 Se				
Correlation coeff. (all/weak) ^‡^	51.99/29.92				
Patterson FOM ^‡^	12.58				
Correlation coeff. (E) ^‡^	0.505				
*R*-cullis ¶ (anom., acentric)	0.843				
Phasing power ¶	0.916				
FOM ** [cos(phase error)] (acentric/centric)	0.2996/0.0781				
Solvent flattening					
*R*-factor ** (before/after)	0.4820/0.2120				
Overall correlation on |E|^2^ ** (before/after)	0.3246/0.8833				
Correlation on |E|^2^/contrast (original/inverted)	0.4688/0.2766				
Refinement					
Reflections	106789 (17167)	106463 (17174)	213700 (41856)	159525 (11143)	47006 (3004)
Reflections *R*_free_	2205 (346)	2209 (349)	2999 (545)	1957 (159)	1911 (130)
*R*-factor ††	0.140 (0.162)	0.146 (0.199)	0.226 (0.281)	0.171 (0.244)	0.200 (0.352)
*R*-free	0.175 (0.207)	0.187 (0.219)	0.250 (0.321)	0.208 (0.275)	0.263 (0.408)
Protein/glycerol/ water/other atoms	9091/42/1619/0	9101/42/1664/0	36241/0/0/0	11299/102/1822/27	12441/6/350/0
Overall B-factor (Å^2^)	20.0	24.9	51.4	25.9	64.8
Ramachandran stats ^‡‡^ (%)	96.5/99.8	96.5/99.6	96.2/99.9	96.7/99.5	95.5/99.9
rmsd ¶¶ bonds (Å)/angles (°)	0.013/1.3	0.013/1.5	0.005/0.9	0.011/1.4	0.011/1.5
PDB code	4UXE	4UXF	4UXG	5NXF	5NXH

* Values in parentheses are for the highest resolution bin (where applicable). † R_symm_ = Ʃ_h_Ʃ_i_|I_hi_ − <I_h_>|/Ʃ_h_Ʃ_i_|I_hi_|, where I_hi_ is the intensity of the *i*th measurement of the same reflection and <I_h_> is the mean observed intensity for that reflection. ^‡^ Found by Shelxd. ¶ Calculated with Autosharp. ** According to Solomon. ^††^ *R* = Ʃ||*F*_obs_(*hkl*)| − |*F*_calc_(hkl)||/Ʃ|*F*_obs_(*hkl*)|. ^‡‡^ According to the program Molprobity. The percentages are indicated of residues in favored and allowed regions of the Ramachandran plot, respectively. ¶¶ Estimates provided by the program Refmac. SeMet: selenomethionine; ESRF: European Synchrotron Radiation Source; PF: Photon Factory; FOM: figure of merit; rmsd: root mean square difference; PDB: Protein Data Bank.
